# Hunters and hunting across indigenous and colonist communities at the forest-agriculture interface: an ethnozoological study from the Peruvian Amazon

**DOI:** 10.1186/s13002-018-0247-2

**Published:** 2018-08-10

**Authors:** Wendy Francesconi, Vincent Bax, Genowefa Blundo-Canto, Simon Willcock, Sandra Cuadros, Martha Vanegas, Marcela Quintero, Carlos A. Torres-Vitolas

**Affiliations:** 1 International Center for Tropical Agriculture, Av. La Molina 1895, La Molina, Lima, Peru; 2 Centre for Interdisciplinary Science and Society Studies, Universidad de Ciencias y Humanidades, Av. Universitaria 5175, Los Olivos, Lima, Peru; 3 Centre for Biological Sciences, University of Southampton, Southampton, SO17 1BJ UK; 4 Universidad Agraria de la Molina, Av. La Molina, Lima, Peru; 5 International Center for Tropical Agriculture, Km 17 Recta Cali-, Palmira, Colombia; 6 Faculty of Social and Human Sciences, University of Southampton, Southampton, SO17 1BJ UK

**Keywords:** Food security, Hunting, Livelihood strategies, Bush meat, Ucayali

## Abstract

**Background:**

Wildlife has been traditionally used by forest communities as a source of protein, and the Peruvian Amazon is no exception. The articulation of colonist and indigenous communities to urban centers and markets results in changes in livelihood strategies and impacts on wildlife populations. To address the threat of overhunting and forest conversion, we provide a generalized characterization of colonist and indigenous communities and their hunting activities near Pucallpa, Ucayali, Peru.

**Methods:**

A semi-structured household survey was conducted to characterize hunters and describe their prey collections. The data were analyzed by conducting a Kruskal-Wallis test, a multiple regression analysis, and by estimating the harvest rate (*H*).

**Results:**

Less wealthy households were more actively engaged in hunting for food security and as a livelihood strategy. Additionally, older hunters were associated with higher hunting rates. Although the percentage of hunters was relatively low, estimated hunting rates suggest overharvesting of wildlife. Lowland pacas (*Cuniculus paca*) were the most frequently hunted prey, followed by red brocket deer (*Mazama americana*) and primates. While hunting intensity was not significantly different between indigenous and colonist communities, hunting rate disparities suggest there are different types of hunters (specialized vs. opportunistic) and that prey composition differs between communities.

**Conclusion:**

Close monitoring of wildlife populations and hunting activities is ideal for more accurately determining the impact of hunting on wildlife population and in turn on forest health. In lack of this type of information, this study provides insight of hunting as a shifting livelihood strategy in a rapidly changing environment at the forest/agriculture frontier.

**Electronic supplementary material:**

The online version of this article (10.1186/s13002-018-0247-2) contains supplementary material, which is available to authorized users.

## Background

Subsistence hunting has traditionally been an essential livelihood strategy for food security and nutritional health in Amazonian communities. However, economic development, population growth, and the construction of roads in the Amazon have transformed the landscape and the livelihoods of native inhabitants and incoming settlers [23, 41]. The encroaching agricultural frontier into forest land has been identified as a major driver of wildlife habitat destruction in the Amazon region [61, 66]. Simultaneously, the increased accessibility to remote wilderness areas facilitates the extraction and commercialization of wildlife and bush meat, leading to the local and global extinction of species [45, 46, 50]. Exacerbated by rapid land use change and fueled by more effective hunting techniques, wildlife populations are being depleted across tropical regions [5, 39, 70, 72].

Amazon deforestation figures in Peru indicate that almost eight million hectares of primary forest have been converted to agriculture and other land uses. This represents approximately 10% of the original forest cover [35]. Seeking better livelihood opportunities through greater natural resource availability and escaping from conflicts and negative climate change impacts, human migration from the Andean highlands to the lowland Amazon basin has been accompanied by the incorporation of agricultural activities and the establishment of new extractive markets and trades [34, 48]. This rapid growth of a natural resource-based economy in the Peruvian Amazon has led to increased pressures on forest areas [2, 6, 13].

Local extinctions of wildlife species have been reported due to overharvesting [4, 10]. The unsustainable collection of game species is known to trigger feedback mechanisms that can negatively affect entire ecosystems and the services they render [71]. Hence, hunting as a livelihood strategy may no longer be sustainable, reliable, or a desirable activity for communities living in a rapidly changing landscape with a fast growing agricultural and/or timber harvesting economy. While both indigenous and colonist populations have been associated with hunting activities, the socioeconomic and cultural factors that underpin or explain adverse hunting behavior are not clearly understood. It is also not clear what the current situation of game species may be in many Amazon territories in the country [4]. Although conservation scientists generally agree that traditional forest management strategies by indigenous communities are more sustainable compared to those adopted by colonists [12, 32, 63], hunting activity remains poorly understood at the forest-agriculture interface [33, 51].

A better understanding of hunter characteristics and hunting activity within a given socioeconomic and cultural context is critical to address the threat of overharvesting, to support knowledge-based decision making, and to formulate responsible wildlife management policies. At the forest-agriculture interface near Pucallpa in the Amazon Department of Ucayali in Peru, we hypothesize that indigenous communities remain more heavily dependent on hunting activities as a livelihood strategy compared to colonist communities. Furthermore, we hypothesize that hunting activities among indigenous communities are more sustainable than those of colonist communities. Motivated by current knowledge gaps regarding hunting activity in Ucayali, we aimed to (1) compare hunting intensity and (2) characterize hunters in colonist and indigenous communities near the city of Pucallpa in the Department of Ucayali in the Central Peruvian Amazon and (3) describe hunting activity and its potential implications on prey viability. While most ethnozoological studies on hunting in the Peruvian Amazon have been carried out in heavily forested or protected areas [20, 26, 38], our study also includes highly deforested and agricultural areas, allowing for the comparison of hunters and hunting activity across a deforestation and cultural gradient in a rapidly changing environment.

## Methods

This study is part of a larger research project called “Attaining Sustainable Services from Ecosystems through Trade-Off Scenarios,” or ASSETS [49], which aims to understand the linkages between ecosystem services, food security, and nutritional health in poor communities at the forest-agricultural interface.

### Study area

As part of a larger and multicountry research project, in Peru, ASSETS was carried out in communities located in the lowland Amazon rainforests in the provinces of Coronel Portillo and Padre Abad within the Department of Ucayali. The region has a typical tropical climate, with monthly temperatures ranging between 20 and 36 °C and precipitation between 1535 and 2100 mm year^−1^. The rainy season usually takes place between the months of February and May [42].

The Department of Ucayali has an estimated urban population of 40%, with approximately 210,000 people living in the capital city Pucallpa [29]. The remaining population can be classified as indigenous or multi-ethnic rural communities (hereafter colonist communities). The department’s observed rapid population growth was prompted by the construction of the Federico Basadre highway in the 1940s [64], which lead to the influx of logging activities and agribusiness [27]. To date, about 37% of the Aguaytía river basin, which is located within the studied provinces, has been deforested since the construction of the highway [6]. As the city of Pucallpa represents the main market in the region, extensive agricultural production takes place in the surrounding areas and along the paved and unpaved road networks. Annual deforestation rates in the Aguaytía basin are estimated at 0.28%, which represents almost 5000 ha of primary forest loss every year [6].

**Fig. 1:**
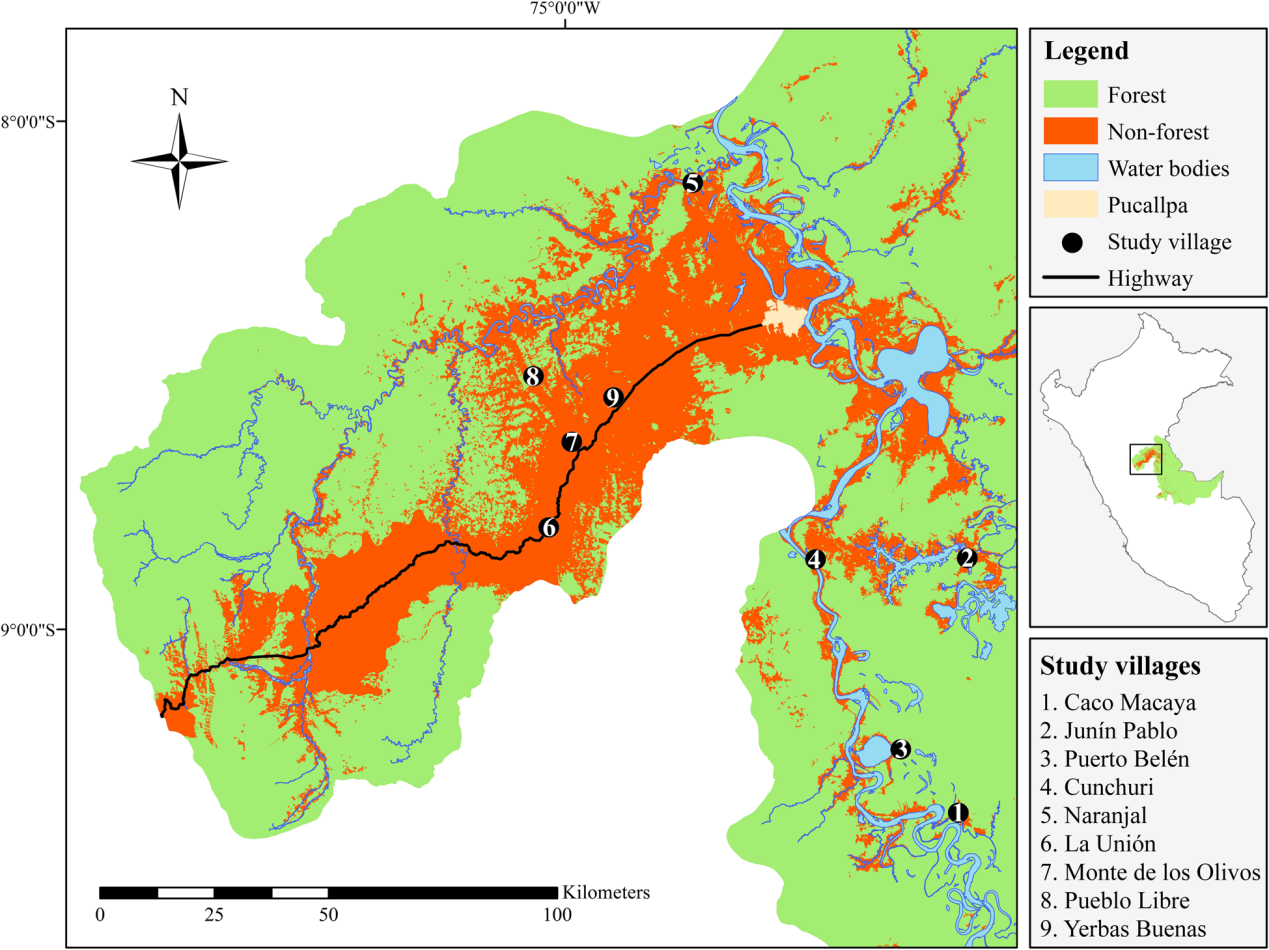
Study area depicting the location of the nine villages interviewed in the Department of Ucayali, Peru

The communities included in this study (Fig. 1; Table 1) can be classified as follows: three indigenous communities (Caco Macaya, Junín Pablo, and Puerto Belén), two riverine colonist communities (Cunchuri and Naranjal), and four land-based colonist communities (La Unión, Monte de los Olivos, Pueblo Libre, and Yerbas Buenas). Colonist communities are comprised of people from varying origins, in particular migrants from Andean highland areas, whereas the indigenous communities are largely composed of native Amazon inhabitants belonging to the Shipibo-conibo ethnic group. In terms of land tenure, indigenous communities tend to have communal titles or similar forms of legal recognition, whereas colonist communities usually have some form of individual property title. Nonetheless, Cronkleton and Larson [14] found that under communal properties, individual access rights and arrangements are often in place, and collective concepts and practices are usually adopted on individual properties in the Peruvian Amazon. In both types of communities, uncertainty exists as per the individual’s land boundaries and rights of use, which can have negative implications on hunting activities.

**Table 1:** Village and survey characteristics

Communities	Population [28]	HH survey sample	Hunter survey sample	Principal livelihood strategies	Approx. area	Access routes	Time to Pucallpa	Primary forest cover
*Indigenous*								
1. Caco Macaya	1031	34	10	Subsistence agriculture (corn, banana, and tubers), fishing, and logging	20 km^2^	River	18 h	Medium
2. Junín Pablo	922	26	3	Subsistence agriculture (corn, cassava, and banana), commercialization of bijao leaves, fishing, and logging	8 km^2^	River	16 h	Medium
3. Puerto Belén	893	35	7	Subsistence and commercial agriculture (banana, papaya, and corn), fishing, cattle ranching, and hunting	11 km^2^	River	12 h	High
*Riverine colonist*								
4. Cunchuri	604	22	3	Subsistence agriculture (fruit trees, small farm animals) fishing and hunting	35 km^2^	River	3 h	Medium
5. Naranjal	289	11	6	Subsistence agriculture (corn, banana, and rice), commercial agriculture (sugar cane, and cacao), logging, fishing, and cattle ranching	10 km^2^	River and road	4 h (river); 1 h (road);	Medium
*Land-based colonist*								
6. La Unión	959	29	7	Subsistence agriculture, commercial agriculture (palm oil and cacao), and cattle ranching	55 km^2^	Road	1.5 h	Medium
7. Monte de los Olivos	313	26	3	Subsistence agriculture and commercial agriculture (palm oil and cacao)	25 km^2^	Road	1.5 h	Low
8. Pueblo Libre	354	13	2	Subsistence agriculture and commercial agriculture (palm oil)	25 km^2^	Road	3 h	Medium
9. Yerbas Buenas	337	34	1	Subsistence agriculture and commercial agriculture (palm oil, pineapple, and citric fruits)	20 km^2^	Road	1 h	Low
Total	5702	230	42		209 km^2^			

To provide context, forest cover values were classified into three levels: “High” represents that 81% or more of the community territory has a 60% canopy cover, “Medium” represents that between 51 to 80% of the territory has a 60% canopy cover, and “Low” represents that 50% or less of the territory has a 60% forest cover

### Data collection

Among the ASSETS project data collection activities, a comprehensive household survey questionnaire composed of 30 modules was developed by an interdisciplinary team of scientists, which was led by the University of Southampton. The survey instrument included multiple choice and open-ended questions (qualitative and quantitative) regarding household characteristics, use of natural resources, livelihood strategies, and dietary habits [58]. From the household survey questionnaire, a subset of 15 questions was extracted to analyze hunting activity and hunter characteristics (Additional file 1: Appendix S1). The interviews in this study were conducted during the rainy season, between February and April 2015, and the households included were randomly selected. The interviews were conducted with the head of the household after official consent was granted by them through specific questions within the survey instrument and previously by local leaders to the ASSETS project team members. To provide context, additional secondary information collected by the ASSETS project was used to describe the communities (Additional file 2: Appendix S2).

### Data analysis

Data analysis focused on (1) the relation between community ethnicity and hunting intensity (number of animals/month), (2) hunter characterization, and (3) hunting activity data inferences (species preferences/availability and potential population viability impacts). To address our first hypothesis, the monthly average number of prey animals reported by hunters was compared based on community type (indigenous, riverine colonist, and land-based colonist) using a Kruskal-Wallis test conducted in R version 3.2.3 (2008). Significant differences in the average number of animals hunted were determined at the 95% confidence interval (*α* = 0.05). In addition, a multiple regression analysis (backwards elimination method) was conducted in SPSS version 20 to determine the characteristics which collectively best reflect hunters in the Ucayali region. The estimated total number of animals hunted per month was set as the response variable, and seven objective and subjective hunter characteristics were used as explanatory variables: age, educational level, ethnicity, wealth (ownership of durable items such as domestic appliances, and materials for labor), number of heads of cattle owned, frequency of fishing, and personal perception of household food security. The variable for wealth was used instead of income as a long-term economic indicator and as a way to estimate the level of comfort in a less biased manner given that income wages may be variable among indigenous communities. Yet, the variable was estimated on a per capita basis for each household. Gender was not considered in the analysis as 95% of the hunters surveyed were male. Categorical explanatory variables were converted to continuous data, and all were ln-transformed to improve the linear fit to the response variable and to ensure normality of the residuals. Given that the number of hunters in the sample was relatively low, the regression model was run to determine predictor variable significance and the type of relation (negative or positive) with the response variable. Standard statistical test procedures (not shown) were carried out to assess the performance of the regression model.

To address our second hypothesis, hunting activity among communities and community types was explored by comparing the types of animals harvested, their relative reproductive strategies, and the potential impacts of hunting in the region. From the interview data, it was not possible to estimate the maximum sustainable yield of commonly hunted species or groups. Instead, we used the harvest rate (*H*) concept proposed by Robinson and Redford [55]. Harvest rate was used to calculate “the number of animals of a species removed by humans per square kilometer in a year.” This was done by taking the total number of animals reported per month for a given species and multiplying by 12 to estimate annual values. Then, it was divided by the aggregated territories of the villages surveyed (209 km^2^). The estimated values for commonly harvested species/taxa were compared to the sustainable harvest values modeled by Robinson and Redford [55] for the same region. While hunting activity reported by the household survey does not fully reflect offtake values (as some hunters may be missing), in the absence of detailed information on species population density, the information collected was used to estimate a preliminary harvest rate and to provide a generalized assessment of current hunting activity and impact.

## Results

### Characteristics of hunters

A total of 230 heads of household were interviewed in the nine communities. Only 18% (*n* = 42) of these households were involved in hunting, who collected at least one prey item per month. The number of hunters per community was as follows: Caco Macaya 10, Puerto Belén 7, La Unión 7, Naranjal 6, Cunchuri 3, Junín Pablo 3, Monte de los Olivos 3, Pueblo Libre 2, and Yerbas Buenas 1 (Table 1). Among these, 80% indicated that wildlife was exclusively used for food. Contrary to our hypothesis and to the finding that indigenous and riverine communities had a higher number of hunters, hunting intensity was not significantly different at the community type level (*p* = 0.675). Most indigenous and colonist hunters collected small quantities of animals (63% reported hunting less than three animals per month). At the same time, both indigenous and colonist communities include individuals who hunted much higher numbers of animals (13% reported hunting more than 10 animals per month). In the indigenous community of Caco Macaya for instance, most hunters collected between one and five animals per month. Yet, there were two hunters who estimated harvesting between 18 and 30 animals per month each.

**Fig. 2:**
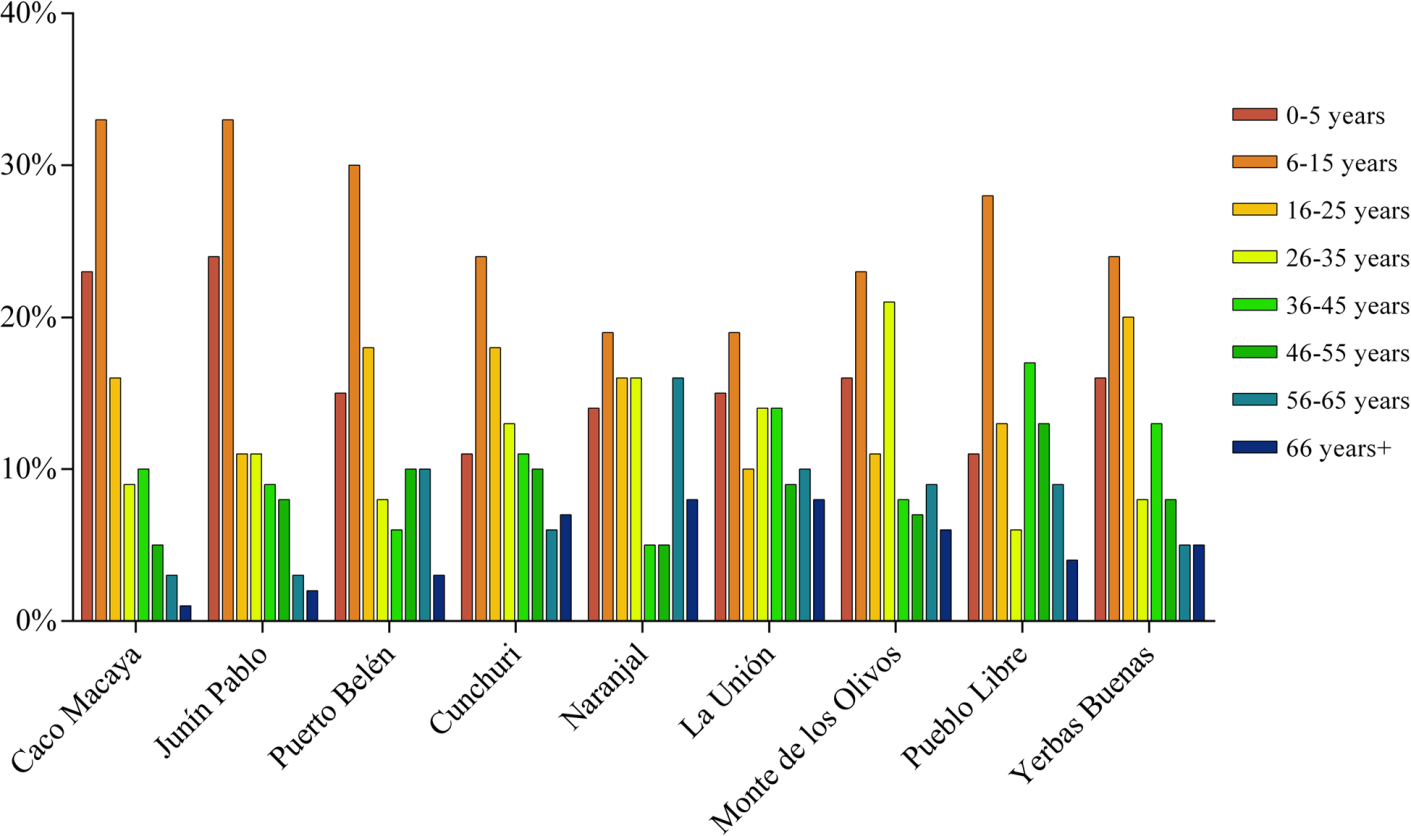
Age distribution per community

According to the multiple regression analysis, only the variables for wealth and age were significant in predicting the number of preyed animals per month, explaining 29% of the variation (adjusted *R*^2^ = 0.288). Wealth, or the number of durable items owned by indigenous and colonist hunters, ranged from 1 to 12 (*M* = 7, SD = 3), and their age varied from 16 to 70 years (*M* = 41, SD = 14). By examining the demographic information collected from the household survey (Fig. 2), a sharp decrease can be noticed in the number of individuals between the ages 16 and 25 (particularly among the indigenous communities). This could likely be interpreted as young individuals leaving their villages, and perhaps moving to urban areas seeking other opportunities. The regression model showed a negative relation between wealth and game takeoff (regression coefficient *β* = − 0.430), and a positive relation between age and hunting success (regression coefficient *β* = 0.374). These results suggest that hunting activities decreased with wealth and increased with age.

### Characteristics of hunting activity

Table 2 presents a summary of game animals as reported by hunters. From the nine communities surveyed, a total of 197 individuals belonging to 18 species or taxa were reported hunted, averaging 4.7 animals/month/hunter. The lowland paca (*Cuniculus paca*) and the armadillo (*Dasypus* sp.) were the most commonly hunted species across all communities. In terms of overall numbers, the lowland paca was also the most harvested game animal, followed by spider monkeys (*Ateles* sp.), armadillos, and brocket deer (*Mazama Americana*). During the rainy season when the data were collected, mammals were the main group hunted in the region. These were followed by birds, while the collection of reptiles was less common. Harvested animals were classified according to their general reproductive productivity (*K* or *r* selection strategies). Species vulnerable to overhunting are typically K-selected, which are those characterized by relatively low reproductive cycles and large body mass [9]. In indigenous communities, 48% of the total number of game animals was r-selected, and 52% was K-selected. In riverine communities, the percentage of r-selected species was slightly higher than K-selected at 54 vs. 46%, respectively. In contrast, land-based communities mostly hunted animals with relatively high reproductive rates (86% r-selected vs. 14% K-selected).

**Table 2:** Description of the prey items reported for the study communities

Scientific name	Common name (Spanish)	Relative reproductive strategy^±^	Community*^∞^	Successful hunting events (no. Indv./month/village)^+^	Harvest rate (*H*) (no. Indv./km/year)^α^
Birds					
*Cairina moschata*	Muscovy duck (*sachapato*)	r	D	1	0.06
*Ortalis gutata*	Speckled chachalaca (*manacaraco*)	K	I	1	0.06
*Penelope* sp.	Guan (*pucacunga*)	K	**B**, I	2	0.12
*Tinamus* sp.	Tinamous (*perdiz*)	r	G, I	2	0.12
Mammals					
*Aotus* sp.	Night monkey (*mono musmuqui*)	K	E, **B**	2	0.12
*Ateles* sp.	Spider monkey (*mono araña negro*)	K	**A**, **B**, **C**, D	22	1.26
*Cuniculus paca*	Lowland paca (*maja*)	r	**A, B**, **C**, D, E, F, G, H, I	77	4.42
*Dasypus* sp.	Armadillo (*carachupa*)	r	**A**, **B**, **C**, D, E, F, G, H, I	19	1.09
*Dasyprocta punctata*	Black agouti (*anuje*)	r	**A**, D, H, I	13	0.75
*Hydrochoerus hydrochaeris*	Capybara (*ronsoco*)	r	**A**	1	0.12
*Mazama americana*	Red brocket deer (*venado*)	K	**B**, **C**, D	18	1.03
*Oreonax flavicauda*	Woolly monkey (*mono choro*)	K	**B**	10	0.57
*Pecari tajacu*	Collared peccary (*sajino*)	K	**B**, **C**, D, G, I	12	0.70
*Saimiri* sp.	Squirrel monkey (*mono huasa*)	K	**B**, D	11	0.63
*Speothos venaticus*	Bush dog (*manko*)	K	E	1	0.06
*Tapirus terrestris*	Lowland tapir (*sachavaca*)	K	**B**	1	0.06
*Tayassu pecari*	White-lipped peccary (*huangana*)	K	**C**	1	0.06
Reptiles					
*Chelonoidis denticulata*	Yellow-footed tortoise (*motelo*)	r	I	3	0.17
Total				197	

^±^Characterization based on Pianka, E.R. [44], and the following taxa-related references: Robinson and Redford [54], Begazo and Bodmer [8], Begazo [7], Peres and Nascimento [47], and Parry et al. [43]

*A: Junín Pablo, **B**: Caco Macaya, **C**: Puerto Belén, D: Naranjal, E: Cunchuri, F: Yerbas Buenas, G: Pueblo Libre, H: Monte de los Olivos, I: La Unión

^∞^Bold letters identify indigenous communities, underlined letters identify riverine colonist communities, and the rest of the letters identify land-based colonist communities

^+^Estimate of animals hunted per month within or nearby the community’s territory (exact data for hunting area is not available)

^α^The total number of individuals for each species or taxa harvested was divided by the aggregated area of the community’s territories per year

At the forest/agricultural interface near Pucallpa, if we extrapolate the total number of pacas harvested per hunter per month to a year, and divide by the sum of the sampled community’s territory, the average harvest rate (*H*) would be 4.42 animals/km^2^/year. By the same standards, the harvest rate for spider monkeys, armadillos, and red brocket deer would be 1.26, 1.09, and 1.03 animals/km^2^/year, respectively. At the community type level and within their respective territories, if we compare the estimated harvest rates for each species, we find that indigenous communities reported higher takeoff values (Table 3). While game diversity was also greater in indigenous communities, higher harvest rates could imply overharvesting by hunters in these communities.

**Table 3:** Total number of prey and estimated harvest rate (*H*)^a^ y community type for frequently hunted species/taxon

Scientific Name	Common Name (Spanish)	Total Indv./Month (Harvest Rate *H*)^b^			Proposed Sustainable Harvest Rate (*H*) by Robinson and Redford (1991)
		Indigenous	River-Based Community	Land-based Community	
*Ateles sp.*	Spider monkey (mono araña negro)	21 (6.46)	1 (0.26)	0 (0.00)	(0.16)
*Cuniculus paca*	Lowland paca (maja)	38 (11.69)	17 (4.53)	22 (2.11)	(1.31)
*Dasypus sp*.	Armadillo (carachupa)	4 (1.23)	4 (1.07)	11 (1.06)	(5.19)
*Mazama americana*	Red brocket deer (venado)	9 (2.76)	9 (2.40)	0 (0.00)	(0.67)

^a^Harvest Rate (*H*) is estimated as the number of individuals. /km^2^/year

^b^The aggregated area for each type of community was: Indigenous= 39 km^2^, River-based= 45km^2^, Land-based= 125km^2^

## Discussion

Although the proportion of households engaged in hunting activities was greater among indigenous communities (Table 1), the average number of animals harvested per month was not significantly different among indigenous, riverine, and land-based colonist communities. Studies from the Neotropical region that explicitly compare hunting characteristics associated with native versus non-native cultural backgrounds are few. One study available is the analysis by Jerozolimski and Peres [30] on game harvests from 31 settlements across Neotropical forests. Their results indicate that colonist and indigenous communities are not significantly different in terms of the number of animals collected, biomass consumption, and relative importance of mammal biomass in the overall harvest of wildlife. Furthermore, they claim that the harvest of prey may be more influenced by the local availability of wildlife stocks rather than cultural aspects. These conclusions are reflected in our results. However, an earlier study by Redford and Robinson [51] identified differences between indigenous and colonist communities. In addition to wildlife availability, they attributed differences in hunting intensity to cultural factors such as hunting tools/methods, taboos/prohibitions, and or “agreed upon” hunting rules within the community. Their analysis was based on 19 studies carried out between 1960 and 1980 in the Neotropics. Yet, more recent research by de Thoisy et al. [18] in French Guiana demonstrates that the ethnic background of hunters did not have a detectable influence on the extent to which prey populations were being harvested. These contrasting outcomes suggest that comparisons in hunting intensity between communities may be context specific in time and space. Given that the communities surveyed in this study represent a current portrayal of hunting activity across the forest/agricultural frontier in Ucayali, the lack of differences could indicate a cultural merging of livelihood activities.

Changes in livelihood activities among indigenous communities through the adoption of logging, agriculture, and the commercialization of non-timber forest products (i.e., bijao leaves) may be displacing hunting as a primary food procurement activity, which agrees with findings elsewhere (e.g., Santos-Fita [57]). While ethnic background and cultural beliefs have been shown to influence hunting behavior [16, 62], in communities near Pucallpa, the relevance of ethnic and/or cultural differences may be quickly shifting due to changes in diet, changes in the environment, and the involvement in commercial activities [15, 25, 56, 67]. Logging and agricultural expansion leads to forest loss and degradation, which in turn leads to wildlife decline [31]. Under this forest loss/degradation—wildlife decline treadmill pattern, hunting could further exacerbate the deterioration of remaining wildlife populations, resulting in the so-called “empty forest” [52]. The lower availability of game becomes a disincentive for hunting activities as hunters need to travel further and spend more time seeking prey.

While most hunters reported to consume the harvested prey, the disparity in hunting intensity among hunters suggests there are two types of engagement in this activity by both indigenous and colonist communities: a more common occasional and opportunistic subsistence hunting, and what could be perceived as commercial hunting. The low number of animals harvested by most hunters suggests a sporadic and opportunistic approach to hunting. For these hunters, prey items may be occasionally harvested while carrying out other activities such as tending to agricultural plots, fishing, and/or collecting other forest products. In contrast, a smaller group of frequent or specialized hunters tends to harvest larger quantities of game. Similar findings were reported by van Vliet et al. [67], who classified hunters at the Brazil-Peru-Colombian border as “diversified” and “specialized” hunters (analogous to “opportunistic” and “specialized” here, respectively). Their classification was based on the proportion of bush meat sold and bush meat consumed, as well as the level of involvement in the bush meat market chain. According to van Vliet et al. [67], specialized hunters spent more time in the forest and used more bullets compared to diversified hunters, which resulted in a higher average game offtake. The proposed specialized vs. opportunistic hunters in our study would fit with the classification suggested by van Vliet et al. and helps to explain the lack of significant differences among communities. Specialized hunters could be more actively engaged in the commercialization of bush meat as a prime livelihood strategy, while non-specialized hunters seem to hunt occasionally and for consumption purposes mainly.

While the relation between household socioeconomics and hunting is not yet fully understood [17, 19, 24, 60], the hunter characterization results suggest that household economics have a significant role in determining hunting intensity. Wealthier households are less engaged in hunting and therefore less dependent on this activity compared to less wealthy households. Similarly, Morsello et al. [37] examined cultural and economic factors as predictors of bushmeat consumption. In terms of wealth, estimated as the value of household assets, they found contrasting results. While bushmeat consumption did not decrease with wealth, preference for eating bushmeat did. In contrast to our sampled communities, their study was conducted in riverbank settlements that were mainly indigenous in origin, and with the exception of fish and chicken, access to animal proteins was limited. As per Fa et al.’s [21] research on wealth and bushmeat consumption, geographic and socioeconomic context will directly influence the relation between these variables. For example, in some urban settings, bushmeat can be a luxury for wealthier households [68], whereas in some rural areas, it can be an opportunistic source of animal protein used by less wealthy households [17, 21], as could be the case within our study’s forest/agricultural interface.

Age was also significant in characterizing hunters, showing that hunters tend to be older rather than young. The relationship between age and hunting success has been previously examined by Walker et al. [69]. Their results showed that harvest rates were maximized around the age of 40, while hunting success was dependent on hunting skills rather than physical strength, which could be associated with youth. In contrast, Sirén et al. [60] demonstrated that when indigenous communities were given the option to choose between chicken wire and guns, younger people preferred hunting equipment. In their case, younger hunters were more active harvesting animals than older ones in their communities. As per our age structure results across communities, sharp reductions in generational turnover (due to death, migration, or other) could partly explain why hunting activity could be associated with older rather than younger community members.

### Characteristics of hunting activity

Animals with both low and high reproductive rates were similarly hunted in indigenous and riverine colonist communities, while mainly high reproductive species/taxa were hunted by land-based communities. This reflects a lower availability of K-selected species in areas that have undergone greater land cover changes. Among land-based colonist communities, La Unión had the highest hunting rates. The higher hunting activity reported for this community may be related to a greater presence of forest cover in the village surroundings compared to the other land-based communities surveyed (except for Pueblo Libre, Table 1). The presence of primary forest fragments provides significantly more animals for hunting compared to younger secondary forests [22]. The potentially higher abundance of game populations near La Unión may also explain the relatively higher number of hunters compared to other land-based colonist communities (two to six times higher). In addition, the interface between agricultural fields and remnant forest areas may lead to crop raiding by wildlife, which is likely an important incentive for pursuing hunting activities [38, 59].

According to Bodmer [9], riverine colonist communities located in the Peruvian Amazon prefer to hunt large-bodied ungulates such as tapirs and peccaries over smaller mammals. This preference seems to exist throughout the Amazon region and across communities [36]. However, the actual harvest of animals reported showed that the frequency of hunting large and slow-reproducing ungulates was low, while smaller and relatively faster reproducing pacas were the most common game collected. This could reflect a decline in preferred game populations near the communities, inducing hunters to collect animals beyond their first choice, as shown by Alves et al. [3] and Mesquita and Barreto [36]. In addition to reproductive strategies and other species-specific factors, land cover change has a dampening effect on the abundance (and thus on harvesting) of certain animal populations [11, 36, 65]. For instance, the collared peccary (*Pecari tajacu*) and white-lipped peccary (*Tayassu pecari*) have relatively medium to fast reproductive characteristics, making them potentially abundant in the landscape and thus readily harvested. Yet, they are known to be susceptible to anthropogenic pressures such as human-induced forest cover change, road density, and the expansion of settlements [1]. Populations of other fast-reproducing species such as agoutis, pacas, and armadillos are more resilient to land cover change, which increases their potential abundance and availability as game [38]. Forest/agricultural landscapes such as the study site are prone to be highly disturbed and deforested [6]. Hence, the number and types of animals harvested reflect the community of species that persist in the landscape.

According to the number of prey reported (Table 2) within the aggregated community territories (Table 1), lowland paca populations could be prone to overharvesting. Robinson and Redford [55] modeled sustainable harvest rates for several Neotropical forest animals including the lowland paca. Their harvest rate for lowland paca was 3 times lower (1.31 animals/km^2^/year) than that reported at the forest/agriculture interface in Ucayali. Given that the actual hunting territories utilized by the communities and hunters were difficult to estimate due to the varying degree of forest cover and fragmentation (see also Renoux and Thoisy [53]), in turn it is difficult to confirm if the monthly paca harvest values reported would be sustainable. However, because almost half of hunting activities usually takes place within the first 500 m of a community’s perimeter [40], we could infer that even with the relatively small number of hunters in the region, current harvesting rates are not sustainable for lowland pacas. Similarly, the populations of spider monkeys also seem vulnerable to depletion. According to Robinson and Redford [55], sustainable harvest rates are particularly low for primate species, ranging from 0.16 to 0.40 animals/km^2^/year. At the Ucayali forest/agriculture interface, the values for spider monkeys are several times higher, jeopardizing their viability in the region.

In further examining harvest rates by community type for frequently hunted species, we can observe that the values reported by indigenous communities are higher compared to riverine or land-based colonist communities. While challenging our second hypothesis, indigenous communities collected a greater variety of species, which reduces the overall hunting pressure exerted on any single population. Yet, according to our results, indigenous communities seem to harvest unsustainable numbers of individuals, in particular slow-reproducing species (Table 3). While the study by Ohl-Schacherer et al. [40] in Peru’s Manu National Park also reported unsustainable offtake levels of prey such as spider monkeys by indigenous populations, they attributed the lack of extinction of this and other species to source sink dynamics within the reserve. Yet, at the forest/agricultural interface in Ucayali, unsustainable hunting management practices combined with forest fragmentation and habitat degradation, could certainly lead to the eventual extinction of game species in the region.

## Conclusions

Livelihoods strategies of colonist and indigenous communities near Pucallpa in Ucayali depend directly and indirectly on the services provided by natural and anthropogenic ecosystems. At the forest/agricultural interface, these services include wildlife as a protein food source and/or as a trading commodity. Given that indigenous communities have traditionally hunted for subsistence purposes, significant differences in hunting intensity between indigenous and colonist communities were expected. Yet, changes in land cover due to logging, agriculture, and the commercialization of non-timber forest products may be shifting livelihood strategies and displacing hunting as a primary food procurement activity among indigenous communities. Hunting as a frequent livelihood strategy was practiced by a few individuals (specialist), while hunting as a complementary (opportunistic) livelihood strategy was more common. Furthermore, higher hunting activity was associated with less wealthy households and with older rather than young individuals across the studied communities.

Irrespective of the lack of difference in hunting intensity between indigenous and colonist communities, the composition of game species varied between communities. Villages with medium to high forest cover, which included indigenous and riverine colonist communities, reported greater game diversity, as well as a greater number of game species with slow reproductive strategies. Estimations on hunting rates indicate that the lowland paca is susceptible to local extinction, despite of its relatively rapid reproductive strategy. Other commonly hunted species such as primates may also be vulnerable to overharvesting given their slower population turnover reproductive characteristics.

As the agriculture frontier expands into forested areas in response to the continuous influx of migrants, population growth, and extractive economies, food procurement and the livelihood strategies of indigenous and colonist communities are expected to adapt. Hunting activities for subsistence purposes may not be attractive to younger generations, nor feasible as the distribution and abundance of game populations declines. As the potential demand for wildlife increases due to the development of market economies in the region, further research on hunter-prey dynamics is necessary to target effective conservation strategies aimed at protecting healthy faunal populations, conserving valuable cultural practices and traditional resource-use knowledge, along with developing inclusive and sustainable livelihoods for future generations.

## Additional files

Additional file 1: Appendix S1. Selection of questions extracted from household survey to be used in regression analysis. (DOCX 60 kb)

Additional file 2: Appendix S2. ASSETS study Peru village characterization. (DOCX 81 kb)
