# Supplementary material for: Hunters and hunting across indigenous and colonist communities at the forest-agriculture interface: an ethnozoological study from the Peruvian Amazon
Source: J Ethnobiol Ethnomed. 2018 Aug 10;14:54. doi: 10.1186/s13002-018-0247-2 (PMC6086032; doi:10.1186/s13002-018-0247-2)
Supplement: Supplementary file 1 — Appendix S1. Selection of questions extracted from household survey to be used in regression analysis. (DOCX 60 kb) [file 13002_2018_247_MOESM1_ESM.docx]

APPENDIX A. SELECTION OF QUESTIONS EXTRACTED FROM HOUSEHOLD SURVEY TO BE USED IN REGRESSION ANALYSIS

Attaining Sustainable Services from Ecosystems through Trade-off

Scenarios (ASSETS)

Peru

Project Code: NE/J002267-1

Start date 30 April, 2012

End date 30 September 2016


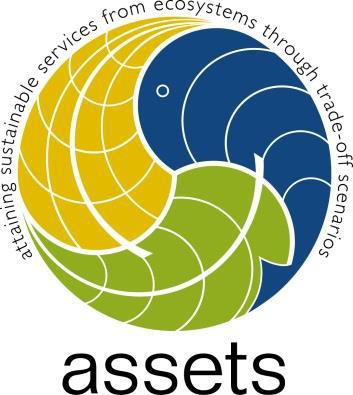


Data extracted from the ASSETS Household Survey Questionnaire

| **No.** | **Module ID** | **Module Description** | **Question Code #** | **Description** |
| --- | --- | --- | --- | --- |
| 1 | ID Info. |  | ID_Community | Community name |
| 2 | ID Info. |  | ID_Household | Home number |
| 3 | A | Household Information | a_1 | Name of head of household |
| 4 | B | Sociodemographic Data | b_1 | List of people in the household |
| 5 | B | Sociodemographic Data | b_4_a | Age of each household member |
| 6 | B | Sociodemographic Data | b_17 | To what ethnic group do you belong (1) Spanish, (2) Shipibo-conibo, (3) Quechua, (9) Other-Specify. |
| 7 | C | Education | c_6 | Education level: From the list what is your highest level of education (0) Preschool, (1) Primary 1st grade, (2) Primary 2nd grade, (3)Primary 3rd grade,(4) Primary 4th grade, (5) Primary 5th grade, (6) Primary 6th grade, (7) Secondary School 1st year (8) Secondary School 2nd year (9) Secondary School 3rd year (10) Secondary School 4th year (11) Secodnary School 5th year, (12) Technical School, (13) University, (14) Graduate School, |
| 10 | J | Food Security | j_2_b | During the last six moths how often the food you had obtained did not last enough and you didn't had the resources to get more: (1) Nunca, (2) Somethimes, (3) Frecuently, (4) No reply, (5) Does not know |
| 9 | L | Goods and Expenses | l_2 | How many of the following durable goods do you possess: (1) Television, (2) Radio, (3)Stereo, (4) Wood or Charcol Stove, (5) Kerosene or Parafine Stove, (6) Gas Stove,(7) Refrigerator,(8) Wood Table,(9) Chairs,(10) Sofa, (11) Beds,(12) DVD / VCR, (13) Computer ,(14) Electric or Diesel Generator,(15) Solar Panels,(16) Satelite TV Antena, (17) Celular Phone, (18) Sowing Machine, (19) Bicycle, (20)Motorcycle,(21) Moto-taxi,(22)Car,(23) Minivan, (24) Canoe,(25) Motor boat in spanish Peque Peque, (26) Large Motor Boat in spanish Pecarari. |
| 11 | AGE | Cattle | AGe_3_a | How many bulls and cows do you currently own |
| 12 | AGH | Fishing | AGh_6 | In the last month, how many weeks you were able to catch fish. |
| 13 | AGI | Hunting | AGi_2 | List the different animals most comonly hunted in the last six months |
| 14 | AGI | Hunting | AGi_8 | What is the average number of animals hunted per month for each especies listed. |
