# Supplementary material for: Hunters and hunting across indigenous and colonist communities at the forest-agriculture interface: an ethnozoological study from the Peruvian Amazon
Source: J Ethnobiol Ethnomed. 2018 Aug 10;14:54. doi: 10.1186/s13002-018-0247-2 (PMC6086032; doi:10.1186/s13002-018-0247-2)
Supplement: Supplementary file 2 — Appendix S2. ASSETS study Peru village characterization. (DOCX 81 kb) [file 13002_2018_247_MOESM2_ESM.docx]

APPENDIX B. ASSETS STUDY PERU VILLAGE CHARACTERIZATION

Attaining Sustainable Services from Ecosystems through Trade-off

Scenarios (ASSETS)

Peru

Project Code: NE/J002267-1

Start date 30 April, 2012

End date 30 September 2016


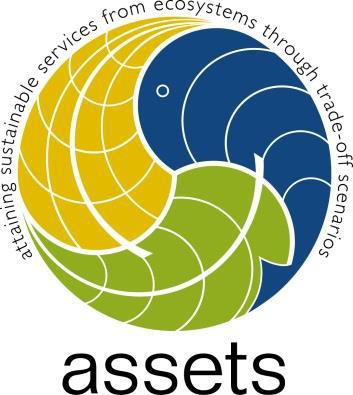


| **Community Name** |  | **Caco Macaya** |
| --- | --- | --- |
| **GPS coordinates** |  | 0576258 E, 8966027 S |
| **Number of HH** |  | 207 |
| **Total population** |  | 1031 |
| **Land use** | **Forest (% in 2014 in Global Forest Change)** | % with 60% canopy within 5 km diameter (excluding oil palm): 71% (Hansen, M.C., Defries, R.S., Townshend, J.R.G., & Sohlberg, R. (2000). Global land cover classification at 1km spatial resolution using a classification tree approach. International journal of remote sensing 21(6-7):1331-1364.) |
|  | **Deforestation rate (% per year)** | % deforestation (2001 - 2014) within a 5km diameter: 17.47 (Global Forest Change). |
| **Agriculture** | **Description** | Subsistence agriculture is the main economic activity. There is livestock production, but it’s a secondary activity for household consumption purposes. Main crops include cassava, plantains, maize and some tubers ("sachapapa", sweet potato, "daledale"). |
| **Soils** | **Description** | Highland areas present abundant, but infertile clay soils. The "restingas" however, present fertile sandy soils, but are scarce. The "barrizal" (clay and mud) areas have high fertility but are scarce and seasonal. Lowlands and beach areas have fertile sandy soils, but are scarce and seasonal. |
| **Natural features** | **Major Rivers** | Ucayali |
|  | **Other** | Conflict with the Puerto Belén community over timber rights. |
| **Distance to market** |  | The city of Pucallpa would be the closest market, though a few local grocery shops are available within the community. The community's port however, may be considered a market by the locals as it is possible to buy/sell fish. |
| **Accessibility** |  | Pucallpa can be reached in 10 hours by private boat, or in 18 hours by public boat. |
| **Village infrastructure** |  | Public school, community center, local canteen, health center and a chapel. |
| **Qualitative descriptions** | **Ecosystem services identified by Women and Men in order of importance.** | Women: water, fish, soil fertility, firewood, wild fruits, wild animals, seeds for crafts, medicinal plants, precipitation, and timber. Men: firewood, medicinal plants, morning dew and rain, productivity of the soil, seeds for craft, wild fruits and timber trees. Regarding trends: medicinal plants, wild animals, firewood, wild fruits, timber, soil productivity, and seeds for crafts will decrease. However, the productivity of lowland soils has been maintained. These trends affect the economy of the community and has induced a change on diets (e.g. bush meat is being replaced by beef meat). |
|  | **Food security** | The most important source of food are home-gardens due to their proximity and cost. A second source is the grocery store due to its accessibility. A third source of food is the market in Pucallpa due to the variety of products it provides. |
|  | **Drivers of change** | 1. Deforestation. 2. Population growth. 3. Poor agriculture practices such as burning. |
|  | **Livelihoods** | Main livelihood activities include agriculture for household consumption, fishing for household consumption and for sale, sale of handicrafts and seeds and daily wages for labour outside of the community. Other complementary activities include: logging, livestock for household consumption, and ownership of grocery store. |
|  | **Other key features** | In 2014 the community was fined because the company Capirona cut trees outside the permitted area declared by the Forest Resources Supervision Agency (OSINFOR). |

| **Community Name** |  | **Junín Pablo** |
| --- | --- | --- |
| **GPS coordinates** |  | 0588371 E 9021625 S |
| **Number of HH** |  | 184 |
| **Total population** |  | 922 |
| **Land use (from ASSETS land cover maps)** | **Forest (% in 2014 in Global Forest Change)** | % with 60% canopy within 5 km diameter (excluding oil palm): 78.6% (Hansen, M.C., Defries, R.S., Townshend, J.R.G., & Sohlberg, R. (2000). Global land cover classification at 1km spatial resolution using a classification tree approach. International journal of remote sensing 21(6-7):1331-1364.) |
|  | **Deforestation rate (% per year)** | % deforestation (2001 - 2014) within a 5km diameter: 7.01 (Global Forest Change). |
| **Agriculture** | **Description** | Crops are cultivated for both subsistence and commercial purposes, while livestock farming is mainly intended for household consumption. Principal crops for household consumption include cassava, plantain and maize. “Bijao” leaves are grown mainly for commercial purposes. |
| **Soils** | **Description** | The highlands present sandy soils while the lowlands present sandy-clay soils. |
| **Natural features** | **Major Rivers** | Ucayali |
|  | **Other** | The lake Junín Pablo is an important source for fish protein. Several other lakes are used for fishing as well (Lake Chauya, Lake Tapasho, Lake Egipto and Lake De cruces) although it is uncertain whether these are located within the community territory. The creation of a regional conservation area (ACR) has led to a reduced governance of natural resources by the community. Further, the invasion of mestizo families (about 20 years ago) has led to conflicts within the community. |
| **Distance to market** |  | Pucallpa is the nearest market. |
| **Accessibility** |  | Pucallpa can be reached in 8 hours by private boat, or in 15 hours by public boat. |
| **Village infrastructure** |  | Public school, community center, health center and a chapel. |
| **Qualitative descriptions** | **Ecosystem services identified by Women and Men in order of importance.** | Women: soil fertility, fish, clean air, precipitation for crop irrigation, medicinal plants, “shebon” leaves, beneficial/edible insects, “bijao” leaves and inputs for handicrafts. Men: “bijao” leaves, soil fertility, fish, “shebon” leaves, timber, basic materials for crafts, medicinal plants and precipitation for crop irrigation. Regarding trends: “shebon” leaves, inputs for crafts, medicinal plants, fish from the lakes, “bijao” leaves and soil fertility will decrease. The decrease in these ecosystem services affects the economy and food security of the households of the community. |
|  | **Food security** | The main food source is the "chacra", given the significant quantity and variety of food products it can provide. A second food source is the lake, as it gives access to a large amount of food for which low monetary investments are needed. The third food source is the home-garden, due to the variety of foods it can provide, the low investments needed and the ease to obtain products from this source. |
|  | **Drivers of change** | 1. Population growth. 2. New markets for wild products (“bijao” leaves). 3. Deforestation. |
|  | **Livelihoods** | The main livelihood activities include subsistence and commercial agriculture, the gathering of “bijao” leaves for commercial purposes, fishing, logging, sale of handicrafts and seeds and day labor. Complementary activities include livestock farming, the gathering of wild fruits (mainly for household consumption) and the ownership of a grocery store. |

| **Community Name** |  | **Puerto Belén** |
| --- | --- | --- |
| **GPS coordinates** |  | 0571921 E, 8979273 S |
| **Number of HH** |  | 176 |
| **Total population** |  | 893 |
| **Land use** | **Forest (% in 2014 in Global Forest Change)** | % with 60% canopy within 5 km diameter (excluding oil palm): 85.4% (Hansen, M.C., Defries, R.S., Townshend, J.R.G., & Sohlberg, R. (2000). Global land cover classification at 1km spatial resolution using a classification tree approach. International journal of remote sensing 21(6-7):1331-1364.) |
|  | **Deforestation rate (% per year)** | % deforestation (2001 - 2014) within a 5 km diameter: 7.90 (Global Forest Change). |
| **Agriculture** | **Description** | Agricultural activities are conducted for household consumption and for commercial purposes. Livestock products are mainly for household consumption. The main crops are: cassava, plantain, papaya and maize. |
| **Soils** | **Description** | Highland areas present sandy-clay soils, and are used mainly for "aguaje" and "ungurahui" palm plantations. The "restinga" areas have black and sandy soils and are mainly used for agriculture. Lowlands are not frequently used for agriculture. |
| **Natural features** | **Major Rivers** | Ucayali |
| **Distance to market** |  | The nearest market is in Pucallpa. |
| **Accessibility** |  | It takes 8 hours by private boat or 12 hours by public boat to reach Pucallpa. |
| **Village infrastructure** |  | Public school, community center, popular canteen, health center, chapel, light tower, elevated tank. |
| **Qualitative descriptions** | **Ecosystem services identified by Women and Men in order of importance.** | Women: fish from the lake, timber from trees, fertile soils of the "restingas, seeds for crafts and medicinal plants. Men: Timber from trees, fish from the lake, medicinal plants, seeds for crafts and fertile soils. Regarding trends: All the ecosystem services mentioned as the most important for men and women are decreasing, especially the seeds for crafts, soil fertility and timber. Reductions in soil fertility is reflected in the low productivity of some crops and therefore a decrease in the economic benefits from selling these crops. |
|  | **Food security** | The most important food source is the lake because if provides food constantly and because the food can be used to trade for other goods. The second source is the "chacra" due to the same reasons as the lake. The third food sources are the local grocery shops because they provide food throughout the year and because it is near to the household area. |
|  | **Drivers of change** | 1. Fishing techniques. 2. Logging companies. 3. Lack of inputs to fertilize the soil. |
|  | **Livelihoods** | The main activities supporting the community are agriculture, fishing, logging, handcrafts and seeds, and daily wages. Complementary activities include livestock rearing, hunting mainly for household consumption, wild fruits gathering for consumption, and owning a grocery shop. |
|  | **Other key features** | The community has agreed to provide forest concessions to logging companies for cutting specific areas of the forest and to receive 26% of the revenues. Yet, the poor administration by the community authorities and the logging company has resulted in a legal fine for cutting outside the permitted area. This has also resulted in conflicts with the neighboring community Caco Macaya over the rights of the timber located at the border between the two community territories. |

| **Community Name** |  | **Cunchuri** |
| --- | --- | --- |
| **GPS coordinates** |  | 0553075 E, 9020100 S |
| **Number of HH** |  | 117 |
| **Total population** |  | 604 |
| **Land use (from ASSETS land cover maps)** | **Forest (% in 2014 in Global Forest Change)** | % with 60% canopy within 5 km diameter (excluding oil palm): 77.4% (Hansen, M.C., Defries, R.S., Townshend, J.R.G., & Sohlberg, R. (2000). Global land cover classification at 1km spatial resolution using a classification tree approach. International journal of remote sensing 21(6-7):1331-1364.) |
|  | **Deforestation rate (% per year)** | % deforestation (2001 - 2014) within a 5km diameter: 4.24 (Global Forest Change). |
| **Agriculture** | **Description** | The main productive activities in the community include subsistence and commercial agriculture along with fishing. Principal crops cultivated for household consumption include: plantain, watermelon, bean, peanut, chiclayo, rice, maize and cacao. Principal commercial crops include plantain and papaya. |
| **Soils** | **Description** | Soils used for agriculture include the black and sandy soils found in the lowlands, and the black and sandy-clay soils found in the “resting” (non-flooded lowlands). |
| **Natural features** | **Major Rivers** | Ucayali |
|  | **Other** | The Communal Reserve El Sira is located in front of the community. This reserve aims to conserve the fauna and flora of the region. |
| **Distance to market** |  | Pucallpa represents the nearest market. |
| **Accessibility** |  | Pucallpa can be reached in 5 hours by private boat, and in 12 hours by public boat. |
| **Village infrastructure** |  | Public school, community center, chapel, light tower and an elevated tank. |
| **Qualitative descriptions** | **Ecosystem services identified by Women and Men in order of importance.** | Women: Soil fertility, timber, fish, medicinal plants and wild animals. Men: Soil fertility, medicinal plants, fish, wild animals and timber. Regarding trends: soil fertility, timber, fish from rivers and lakes and wild animals will decrease, while the availability of medicinal plants will persist. The decrease in soil fertility resulted in a scarcity of good farmlands, forcing some of the community members to work as day laborer on lands owned by others. Also, the decrease in other ecosystem services has caused economic losses. |
|  | **Food security** | The market represents the principal food source, as it provides a variety of food products year-round. The second source is the grocery store within the community, because of its accessibility. The third source includes the "chacra", followed by the river due to their proximity and the low economic capital required to obtain products. |
|  | **Drivers of change** | 1. Population growth. 2. Logging companies. 3. Overfishing and use of traps for fishing. 4. Deforestation. |
|  | **Livelihoods** | The main productive activities include agriculture and fishing for subsistence as well as commercial purposes. The production of livestock is complementary to these activities. Some community members own a grocery store and others work as day laborer in the agricultural and/or fishing sector. |

| **Community Name** |  | **Naranjal** |
| --- | --- | --- |
| **GPS coordinates** |  | 0518442 E, 9098668 S |
| **Number of HH** |  | 41 |
| **Total population** |  | 289 |
| **Land use (from ASSETS land cover maps)** | **Forest (% in 2014 in Global Forest Change)** | % with 60% canopy within 5 km diameter (excluding oil palm): 79.1% (Hansen, M.C., Defries, R.S., Townshend, J.R.G., & Sohlberg, R. (2000). Global land cover classification at 1km spatial resolution using a classification tree approach. International journal of remote sensing 21(6-7):1331-1364.) |
|  | **Deforestation rate (% per year)** | % deforestation (2001 - 2014) within a 5km diameter: 11.76 (Global Forest Change). |
| **Agriculture** | **Description** | The size of the agricultural fields varies from 2 to 10 ha. They are located at approximately 1 kilometer from the village center. The crops are rain-fed and maintenance of agricultural fields only consists of weeding. The crops cultivated in the village area include cassava, citrus fruits (orange and lemon), bolaina, rice, maize, avocado, chiclayo and watermelon. Main commercial crops include cacao and sugar cane. Main subsistence crops include cassava, plantain, rice and maize. |
| **Soils** | **Description** | Soils used for agriculture include sandy and black soils. They are naturally fertile so there is no need for fertilization. |
| **Natural features** | **Major Rivers** | Aguaytía |
|  | **Other** | The community contains approximately 400 ha of forest. However, there is a conflict about territory with the community El Caribe, whose inhabitants want to exploit the forest. The inhabitants of Naranjal intend to implement oil palm crops in the area where the forest is currently located. |
| **Distance to market** |  | The nearest market is the Nueva Requeña market. It can be reached in 4 or 5 hours by boat, or in 1 hour by car if the road is in good conditions. |
| **Accessibility** |  | Pucallpa can be reached in 2 hours by car (during summertime) and in 1 hour by car + 4 hours by boat (during wintertime). |
| **Village infrastructure** |  | Public school, health center and two water wells. |
| **Qualitative descriptions** | **Ecosystem services identified by Women and Men in order of importance.** | Women: Fish from the stream, fertile soils, sun, precipitation for crop irrigation, timber and river fish. Men: fertile soils, timber, river fish, water from the river, sun and precipitation for crop irrigation. Regarding trends: river water level, timber, fertile soils and river fish will decrease, while sun will increase. These changes will affect their health, water availability, agricultural production and food availability. |
|  | **Food security** | The main food source is the market due to the availability of a wide variety of food products. The second most important source is the "chacra" mainly as it provides stable foods for a low cost. The third source is the grocery store. People perceive that wild foods are less available than 30 years ago, and they think that in the future there will be even less. |
|  | **Drivers of change** | 1. Illegal extraction of timber. 2. Unsustainable fishing strategies and lack of regulations commissioned by the fishing industry. 3. Use of agrochemicals. |
|  | **Livelihoods** | Main activities include agriculture (for household consumption and commercial purposes), logging (“bolaina”) and day labour in agriculture. Other activities are fishing and keeping farm animals (for household consumption and commercial purposes). |

| **Community Name** |  | **La Unión** |
| --- | --- | --- |
| **GPS coordinates** |  | 0498260 E, 9032670 S |
| **Number of HH** |  | 145 |
| **Total population** |  | 959 |
| **Land use** | **Forest (% in 2014 in Global Forest Change)** | % with 60% canopy within 5 km diameter (excluding oil palm): 50.3% (Hansen, M.C., Defries, R.S., Townshend, J.R.G., & Sohlberg, R. (2000). Global land cover classification at 1km spatial resolution using a classification tree approach. International journal of remote sensing 21(6-7):1331-1364.) |
|  | **Deforestation rate (% per year)** | % deforestation (2001 - 2014) within a 5km diameter: 19.62 (Global Forest Change). |
| **Agriculture** | **Description** | Subsistence agriculture is the main activity. Cattle ranching however, is also an important activity in the community. The main crops for household consumption are cassava, maize, plantain and rice. Commercial crops are mainly palm oil and cacao. |
| **Soils** | **Description** | Black and sandy clay soils are used primarily for agriculture. Acid and hard soils are sometimes used for agriculture, but they are nutrient poor. Wetlands are used for "aguajales" and as pastures. |
| **Natural features** | **Other** | This community has an area of approximately 5500 hectares, and is located next to the Federico Basadre highway between kilometer 73 and 78. |
| **Distance to market** |  | The nearest market is the San Juan market. To get there it takes 15 minutes by foot. However, other markets include: (1) Neshuya market, to get there it takes 45 min by mototaxi, (2) von Humbolt market, to get there it takes 45 min by mototaxi, and (3) Pucallpa market, but community members do not go there very often. |
| **Accessibility** |  | 1.5 hours by road to Pucallpa. |
| **Village infrastructure** |  | Public school, community center, health center, chapel and elevated water tank. |
| **Qualitative descriptions** | **Ecosystem services identified by Women and Men in order of importance.** | Women: Soil fertility, precipitation, river fish, wild animals, natural grasslands, medicinal plants and timber trees. Men: water, soil fertility, medicinal plants, river fish and timber trees. Regarding trends: precipitation, medicinal plants, timber trees, stream water to feed the cattle, natural grasses and the "aguaje" have decreased. Some of these changes have negative impacts on cattle ranching activities. Furthermore, the prices of timber as a building material have increased affecting the economy of the households. |
|  | **Food security** | The most important food source is the "chacra". The second source is the grocery shop as it is frequently used. A third food source is the home-garden because it produces different types of products and is near the house area. |
|  | **Drivers of change** | Deforestation for agricultural expansion. |
|  | **Livelihoods** | The principal livelihood activity is agriculture. This activity provides products to sell and for household consumption. Other complementary activities include: logging and sale of timber; ownership of a grocery store; hunting (mainly for sale); gathering of wild fruits (aguaje); fishing; daily wages by labours in agriculture and logging activities. |

| **Community Name** |  | **Monte de los Olivos** |
| --- | --- | --- |
| **GPS coordinates** |  | 0499918 E, 9049657 S |
| **Number of HH** |  | 47 |
| **Total population** |  | 313 |
| **Land use (from ASSETS land cover maps)** | **Forest (% in 2014 in Global Forest Change)** | % with 60% canopy within 5 km diameter (excluding oil palm): 42.7% (Hansen, M.C., Defries, R.S., Townshend, J.R.G., & Sohlberg, R. (2000). Global land cover classification at 1km spatial resolution using a classification tree approach. International journal of remote sensing 21(6-7):1331-1364.) |
|  | **Deforestation rate (% per year)** | % deforestation (2001 - 2014) within a 5 km diameter: 31.95 (Global Forest Change). |
| **Agriculture** | **Description** | Subsistence crops primarily include maize, plantain, cassava and rice, while commercial crops include oil palm and cacao. Oil palm is grown in a monoculture, while the cultivation of cacao coincides with the production of other crops, such as plantain. |
| **Soils** | **Description** | Fertile soils used for agriculture include black soils. On the other hand, red-colored soils are used for oil palm plantations and clay soils are used for cattle farming. |
| **Natural features** | **Major Rivers** | N/A |
| **Distance to market** |  | The nearest market is Neshuya’s market, located 7 km away from the community. In can be reached in 20 minutes by motorbike, car or mototaxi. |
| **Accessibility** |  | Pucallpa can be reached via the road in 1.5 hours. |
| **Village infrastructure** |  | Public school, community center, health center, chapel, elevated tank and the APAIMO (association of oil palm producers) meeting place. |
| **Qualitative descriptions** | **Ecosystem services identified by Women and Men in order of importance.** | Women: rain, river fish, wild animals, timber trees, medicinal plants, wild fruits. Men: not evaluated. Regarding trends: not evaluated. |
|  | **Food security** | Not evaluated |
|  | **Drivers of change** | Not evaluated |
|  | **Livelihoods** | Main livelihood activities include oil palm production, agriculture (for household consumption and commercial purposes), and day labour in oil palm plantations. Complementary activities are associated with the production of fish using fish ponds, transport, and work in grocery stores and in the oil palm factory. |

| **Community Name** |  | **Pueblo Libre** |
| --- | --- | --- |
| **GPS coordinates** |  | 0491680 E, 9060674 S |
| **Number of HH** |  | 76 |
| **Total population** |  | 354 |
| **Land use (from ASSETS land cover maps)** | **Forest (% in 2014 in Global Forest Change)** | % with 60% canopy within 5 km diameter (excluding oil palm): 50.2% (Hansen, M.C., Defries, R.S., Townshend, J.R.G., & Sohlberg, R. (2000). Global land cover classification at 1km spatial resolution using a classification tree approach. International journal of remote sensing 21(6-7):1331-1364.) |
|  | **Forest (ha per capita)** | Unknown |
|  | **Deforestation rate (ha per year)** | Unknown |
|  | **Deforestation rate (% per year)** | % deforestation (2001 - 2014) within a 5km diameter: 36.24 (Global Forest Change) |
| **Agriculture** | **Description** | Main subsistence crops include maize, plantain, cassava and rice. Commercial crops include: oil palm and cacao. All crops are rain-fed. Changes in the climate and the occurrence of plagues are the main issues that affect crop productivity. |
| **Soils** | **Description** | Black soils are nutrient rich and therefore used for agriculture. On the other hand, also white and clay soils are used for agriculture, however fertilization is required as they tend to have a very low production. |
| **Natural features** | **Major Rivers** | N/A |
|  | **Other** | This community is located on the side of the road between Neshuya and Curimaná. This is an important road that leads to the principal cities in the region such as Pucallpa, Curimaná and Aguaytía. |
| **Distance to market** |  | The nearest market is in Curimaná. It can be reached in 30 min by motorcar or car. |
| **Accessibility** |  | It takes 2 hours by road to reach Pucallpa. |
| **Village infrastructure** |  | Public school, health center, chapel, water well and a telecentre. |
| **Qualitative descriptions** | **Ecosystem services identified by Women and Men in order of importance.** | Women: Not evaluated. Men: water from the streams, precipitation for crop irrigation, wild fruits, wild animals, medicinal plants and timber. Regarding trends: Wild animals, stream fish, wild fruits, timber and medicinal plants will decrease in the future. In the case of wild animals, they will completely disappear. The decrease of ecosystem services has affected the economy and food consumption of the families. For example, instead of bush meat they are now consuming frozen chicken, which is perceived as less nutritive for children. |
|  | **Food security** | The main food source is the "chacra", due to its proximity and the availability of food throughout the year. The second food source is the stream due to its proximity and good accessibility during the summer. The third food source is the forest, also because of its good accessibility during the summer. People perceive that wild foods are scarcer than 30 years ago. This trend of decreased availability is expected to continue in the future, except for the cocona; a wild fruit that is starting to be commercialized. |
|  | **Drivers of change** | 1. Deforestation. 2. Over-hunting. 3. Lack of knowledge of good practices to collect wild fruits (palms). |
|  | **Livelihoods** | Main livelihood activities include oil palm production, agriculture (for household consumption and commercial purposes), and day labour in oil palm plantations. Complementary wages are associated with activities in transport, grocery stores and in the oil palm factory. |

| **Community Name** |  | **Yerbas Buenas** |
| --- | --- | --- |
| **GPS Coordinates** |  | 0514852 E, 9062473 S |
| **Number of HH** |  | 84 |
| **Total Population** |  | 682 |
| **Land use** | **Forest (% in 2014 in Global Forest Change)** | % with 60% canopy within 5 km diameter (excluding oil palm): 39.5% (Hansen, M.C., Defries, R.S., Townshend, J.R.G., & Sohlberg, R. (2000). Global land cover classification at 1km spatial resolution using a classification tree approach. International journal of remote sensing 21(6-7):1331-1364.) |
|  | **Deforestation rate (% per year)** | % deforestation (2001 - 2014) within a 5 km diameter was: 16.78 (Global Forest Change). |
| **Agriculture** | **Description** | Agriculture is the main activity for the household consumption and commercial purposes. Livestock farming is a complementary activity. The main crops for the household consumption are plantains, cassava, maize and rice. For commercial purpose, they grow palm oil, pineapple and citrus fruits (mandarin, orange, and lemon). |
| **Soils** | **Description** | The black- clay soil types are a little fertile. The clay soil types are very little fertile. The sandy soil types are the most fertile but on a very thin layer. |
| **Natural features** | **Major Rivers** | N/A |
|  | **Other** | Private henhouses present in the community perimeter are polluting the water sources and the activity has a high consumption of the available sources of water for the community. |
| **Distance to market** |  | The nearest market is Campoverde, which can be reached in 20 min by motorcar or 1 hour by foot. |
| **Accessibility** |  | Approximately 1 hour by road. |
| **Village infrastructure** |  | Public school, local canteen, health center, chapel, elevated water tank. |
| **Qualitative descriptions** | **Ecosystem services identified by Women and Men in order of importance.** | Women: Precipitation, natural fertilizer, productivity of the land, aguajal, timber and firewood from the forest. Men: Precipitation for crop irrigation, productivity of the land, natural fertilizer, firewood and aguajal. Regarding trends: All the ecosystem services mentioned will decrease. Productivity of the land and natural fertilizer affect the agricultural capacity of the community. |
|  | **Food security** | The most important food source is the market due to the availability of food throughout the year, the affordable prices and due to the quantity of foods it offers. The second source is the grocery shop due the quantity and quality of the products that it provides. The third source is the "chacra" due to its accessibility throughout the year and the quantity of food products available. |
|  | **Drivers of change** | 1. Population growth. 2. Lack of community organization. 3. Deforestation. 4. Lack of waste management. |
|  | **Livelihoods** | Agriculture is the main activity supporting the livelihoods of the community. Some complementary activities include: ownership of grocery store; gathering of "aguaje"; daily wages from working in agricultural sector. |
